# Supplementary material for: Selenium substitution for dielectric constant improvement and hole-transfer acceleration in non-fullerene organic solar cells
Source: Nat Commun. 2024 Mar 7;15:2103. doi: 10.1038/s41467-024-46352-2 (PMC10920633; doi:10.1038/s41467-024-46352-2)
Supplement: Supplementary file 1 — Supplementary Information [file 41467_2024_46352_MOESM1_ESM.pdf]

Supplementary Information

Selenium Substitution for Dielectric Constant Improvement and Hole-transfer Acceleration in Non-fullerene Organic Solar Cells

Xinjun He[1,#], Feng Qi[2,3,4#], Xinhui Zou[5,#], Yanxun Li[2,3,6], Heng Liu[7], Xinhui Lu[7], Kam Sing Wong[5,*], Alex K.-Y. Jen[2,3,6,8*], Wallace C. H. Choy[1,*]

[1]Department of Electrical and Electronic Engineering, The University of Hong Kong, Pokfulam Road, Hong Kong SAR, China

[2]Department of Chemistry, City University of Hong Kong, Kowloon, Hong Kong

[3]Hong Kong Institute for Clean Energy (HKICE), City University of Hong Kong, Kowloon, Hong Kong

[4]College of Materials Science and Engineering, Qingdao University, Qingdao, 266071 P. R. China

[5]Department of Physics and William Mong Institute of Nano Science and Technology, The Hong Kong University of Science and Technology, Clear Water Bay, Hong Kong SAR, China

[6]Department of Materials Science and Engineering, City University of Hong Kong, Kowloon, Hong Kong

[7]Department of Physics, Chinese University of Hong Kong, New Territories, Hong Kong SAR, China

[8]Department of Materials Science and Engineering, University of Washington, Seattle, WA, USA.

[#]These authors contributed equally: Xinjun He, Feng Qi, Xinhui Zou

*E-mail: phkswong@ust.hk; alexjen@cityu.edu.hk; chchoy@eee.hku.hk

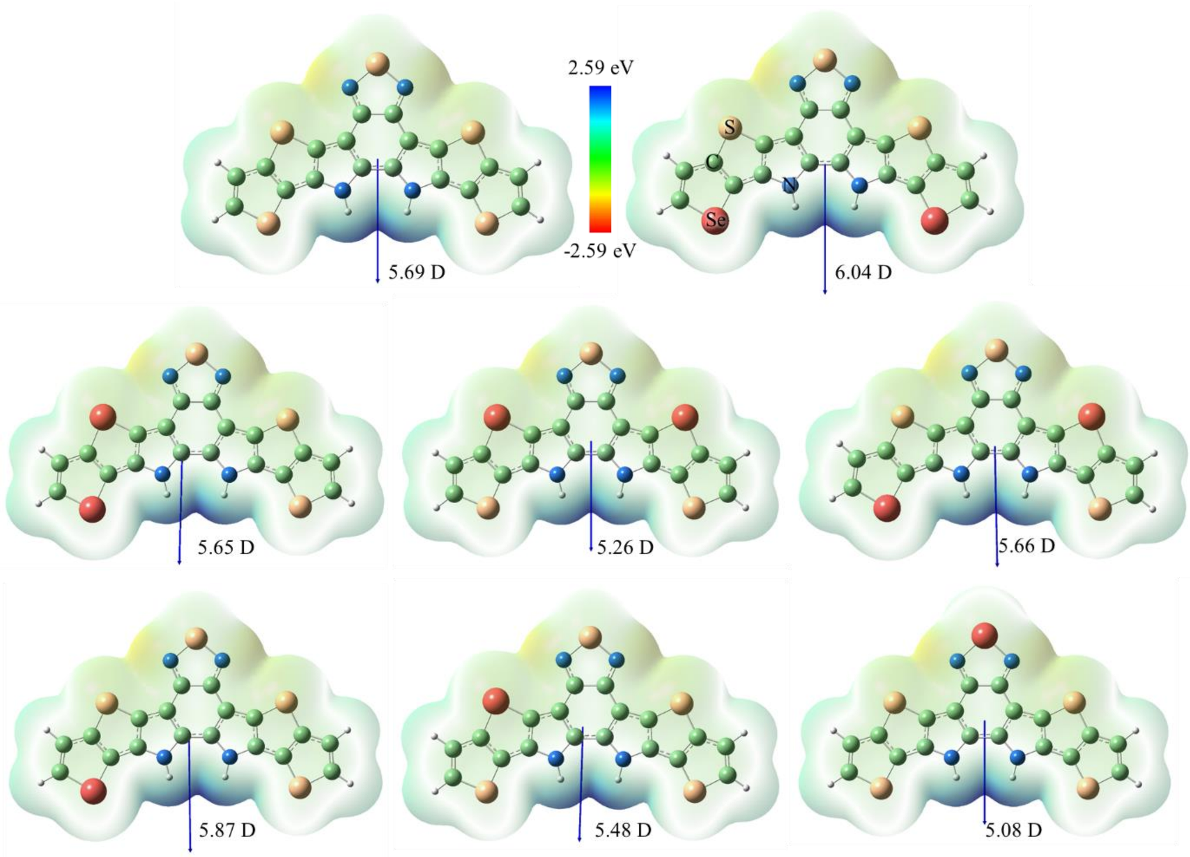

Supplementary Fig. 1. **Electrostatic surface potential contour of central core with Se substituted on different positions**. The calculation is based on the optimized structure at B3LYP/6-31G(d, p) level.

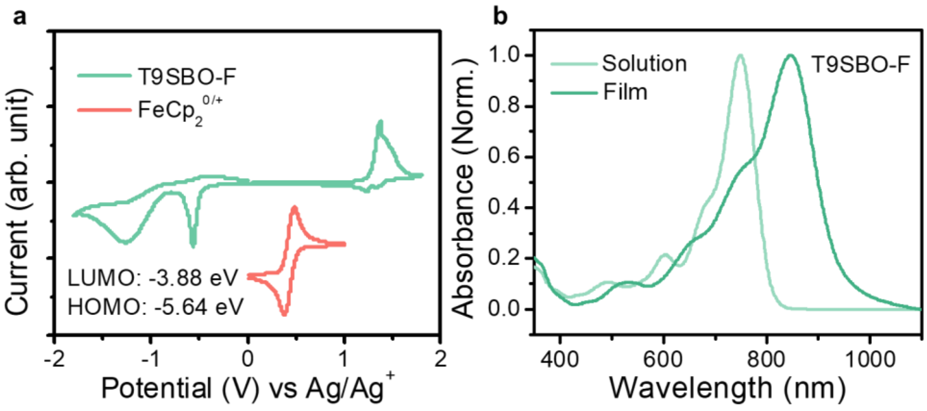

Supplementary Fig. 2. **Optical properties and cyclic voltammetry measurement of T9SBO-F.** (a) UV-Vis absorption spectra and (b) cyclic voltammetry results.

Supplementary Note I

The dielectric constant is measured by impedance spectroscopy following literature by looking at the layered device as a parallel-plate capacitor.[1-3] The device structure is ITO/PEDOT:PSS/test film/PNDIT-F3N/Ag. The real ($\varepsilon'(\omega)$) and imaginary $\varepsilon''(\omega)$ part of the dielectric permittivity can be calculated by following equations.

$$\varepsilon'(\omega) = \frac{Z''(\omega)}{\omega C_0(Z'(\omega)^2+Z''(\omega)^2)} \quad (1)$$

$$\varepsilon''(\omega) = \frac{Z'(\omega)}{\omega C_0(Z'(\omega)^2+Z''(\omega)^2)} \quad (2)$$

$$C_0 = \frac{\varepsilon_0 A}{d} \quad (3)$$

ω is the angular frequency, Z' and Z" are the real and imaginary parts of conjugate complex impedance measured from impedance spectroscopy. EIS is measured on an electrochemical station (Zahner, Zennium Pro) with a frequency range from 10^3 Hz to 10^5 Hz. The measurement is conducted under dark at zero bias to minimize any electrochemical reactions at interfaces.

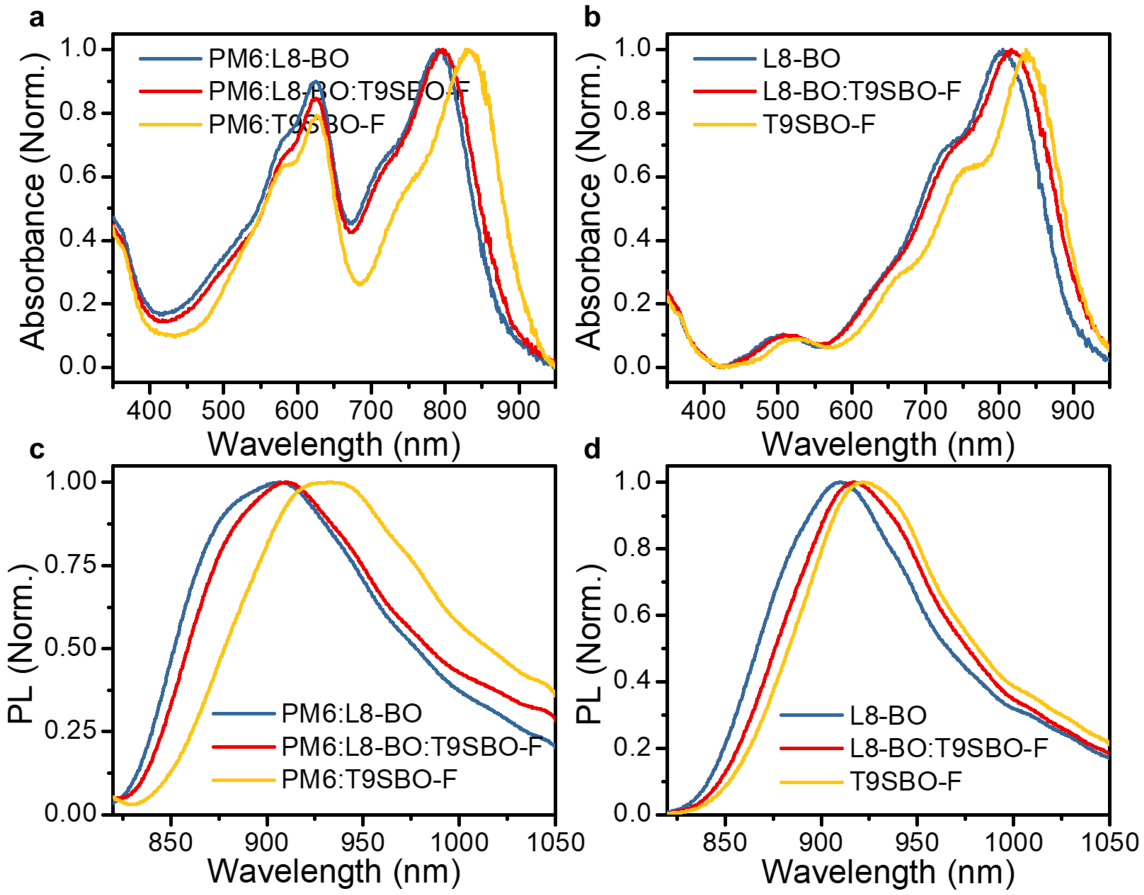

Supplementary Fig. 3. **Optical properties of the blend and neat films**. Absorption spectra of (a) blend and (b) neat acceptor films; PL spectra of (c) blend and (d) neat acceptor films. The L8-BO:T9SBO-F ratio is 1:0.2.

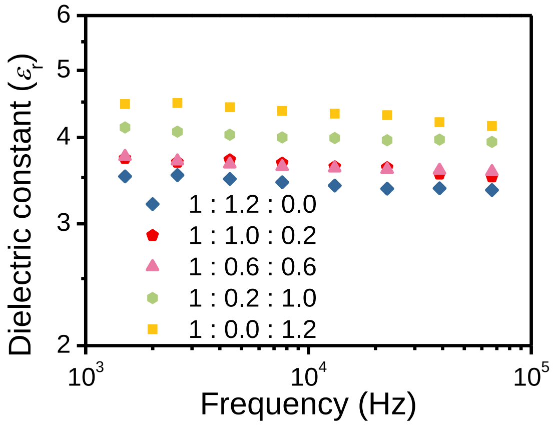

Supplementary Fig. 4. ***ε**$_r$* **of the blends with different Se-NFA ratios**. The legend in the Figure is PM6:L8-BO:T9SBO-F mass ratio.

Supplementary Note II

The hole mobility (μ_h) and electron mobility (μ_e) are measured by space-charge-limited current (SCLC) measurement. The hole-only device structure is ITO/PEDOT:PSS/Active layer/MoO_3/Ag and the electron-only device structure is ITO/ZnO/Active layer/PNDIT-F3N/Ag. By fitting the dark current of the carrier-only devices according to the equation: $J = 9\varepsilon_0\varepsilon_r\mu V^2/8d^3$, where ε_0 is the vacuum permittivity and d is the active layer thickness, we are able to determine the carrier mobility.

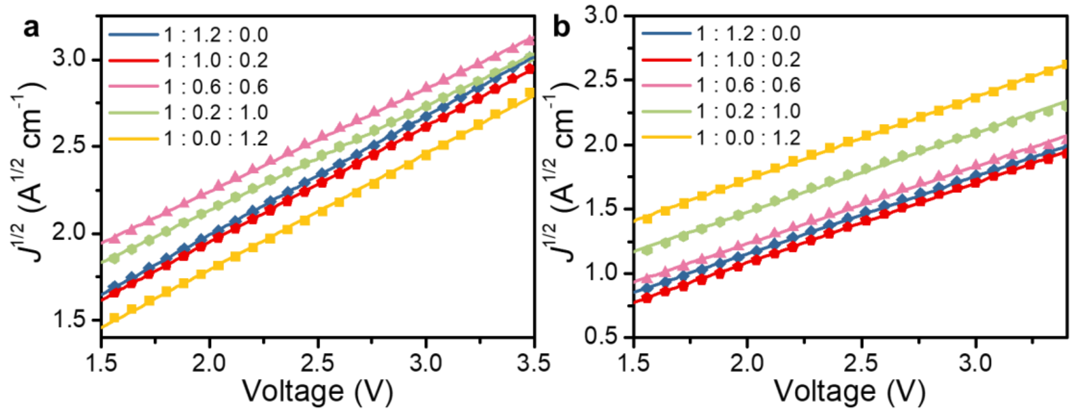

Supplementary Fig. 5. **Carrier mobility measurement**. The $J^{1/2}$-V curves of (a) hole-only and (b) electron-only devices.

Supplementary Table 1. **Electrical properties of devices with different T9SBO-F ratios**. The $J^{1/2}$-V curves of (a) hole-only and (b) electron-only devices.

PM6:L8-BO:T9SBO-F	ε_r	μ_h (cm^2 V^{-1} s^{-1})	μ_e (cm^2 V^{-1} s^{-1})	μ_h/μ_e	R_s (Ω)	R_{sh} (Ω)
1:1.2:0	3.36	10.37	10.69	0.97	70.2	50745
1:1:0.2	3.51	10.07	10.87	0.93	65.8	71827
1:0.6:0.6	3.56	10.74	10.12	1.06	69.3	51901
1:0.2:1	3.93	10.27	9.66	1.06	66.2	58055
1:0:1.2	4.15	10.78	9.97	1.08	63.4	52945

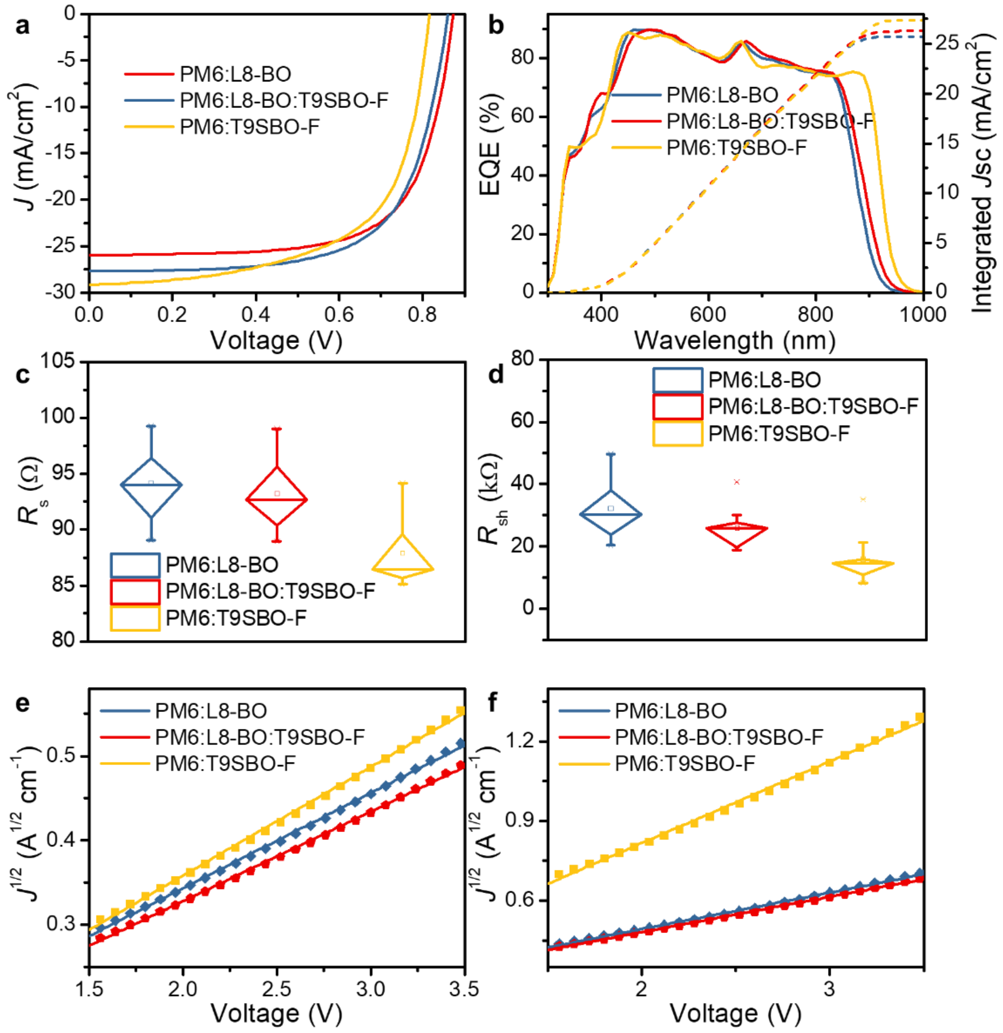

Supplementary Fig. 6. **Device performance and electrical properties of thick OSC (active layer ~300 nm)**. (a) *J-V* curve, (b) EQE spectra, (c) SCLC hole-only device, (d) SCLC electron-only device, (e) device series resistance and (f) device shunt resistance. The ratio of PM6:L8-BO:T9SBO-F thick device is optimized at 1:1:0.2.

Supplementary Table 2. Device performance and electrical properties of thick OSC (active layer ~300 nm).

	Thickness (nm)	J_{SC} (mA/cm^2)	$J_{SC, EQE}$ (mA/cm^2)	V_{OC} (V)	FF (%)	PCE (%)	μ_h (cm^2 V^{-1} s^{-1})	μ_e (cm^2 V^{-1} s^{-1})	μ_h/μ_e	R_s (Ω)	R_{sh} (Ω)
PM6:L8-BO	295 ± 7	26.0 (25.8 ± 0.2)	25.7	0.874 (0.878 ± 0.003)	69.0 (68.2 ± 0.4)	15.7 (15.4 ± 0.1)	8.37	12.44	0.67	94.1	32197
PM6:L8-BO:T9SBO-F	298 ± 5	27.7 (27.4 ± 0.3)	26.3	0.860 (0.861 ± 0.003)	66.9 (66.6 ± 0.5)	16.0 (15.7 ± 0.2)	8.69	13.63	0.64	93.2	26002
PM6:T9SBO-F	293 ± 6	29.1 (29.0 ± 0.2)	27.4	0.816 (0.816 ± 0.001)	62.5 (62.1 ± 0.5)	14.9 (14.7 ± 0.1)	7.12	40.71	0.17	87.9	15970

Supplementary Note III

Ultra-fast femtosecond transient absorption pump-probe spectroscopy was recorded with the excitation of a Ti:sapphire regenerative amplifier femtosecond laser (Coherent Legend). The laser held a fundamental emission of 800 nm at 1 KHz and 100fs pulses, which pumps an optical parametric amplifier (Coheret Opera Solo) for the generation of pulse at 800 or 550 nm to excite acceptors and donor, respectively. The pump beam was chopped at 500 Hz. The probe beam was generated from the output of the amplifier, which is set on a mechanical delay line and focused on a YAG crystal to produce a broadband continuum. The pump and probe beams were overlapped spatially and temporally on the samples encapsulated by a thin quartz slide. To obtain the $\Delta T/T$ signal, a spectrometer (Acton 275), equipped with a line array CCD camera synchronised to the chopper, was employed to measure the transmission.

Upon excitation of the acceptors at 800 nm, the samples present an initial bleach band at around 800-850 nm together with an excited-state absorption (ESA) signal and 900-950 nm due to local-exciton (LE). Following that, a broad ESA signal at 1300-1500 nm emerges, which increases in the first ~250 fs and then decay. This ESA signal serves as intermediate state and shows a second-stage spectral transfer with the onset of a bleach signal at 640 nm, a negative signal at 750 nm and another ESA signal at 1000 nm. The bleach signal at 640 nm coincides with the absorption peak of PM6, which can be ascribed to the hole-transfer from the acceptors to PM6. The negative signal at 750 nm is ascribed to the electro-absorption (EA) signature induced by the electric field change during charge separation (CS) process. The other emerged ESA signal at 1000 nm is due to the photo-induced absorption from positive polarons. In short summary, upon optical pumping the organic blends at 800 nm, an LE signal (900-950 nm) first emerges, followed by an intermediate delocalized state (1300-1500 nm) and finally separated charge signal (CS, 750 nm) appears.

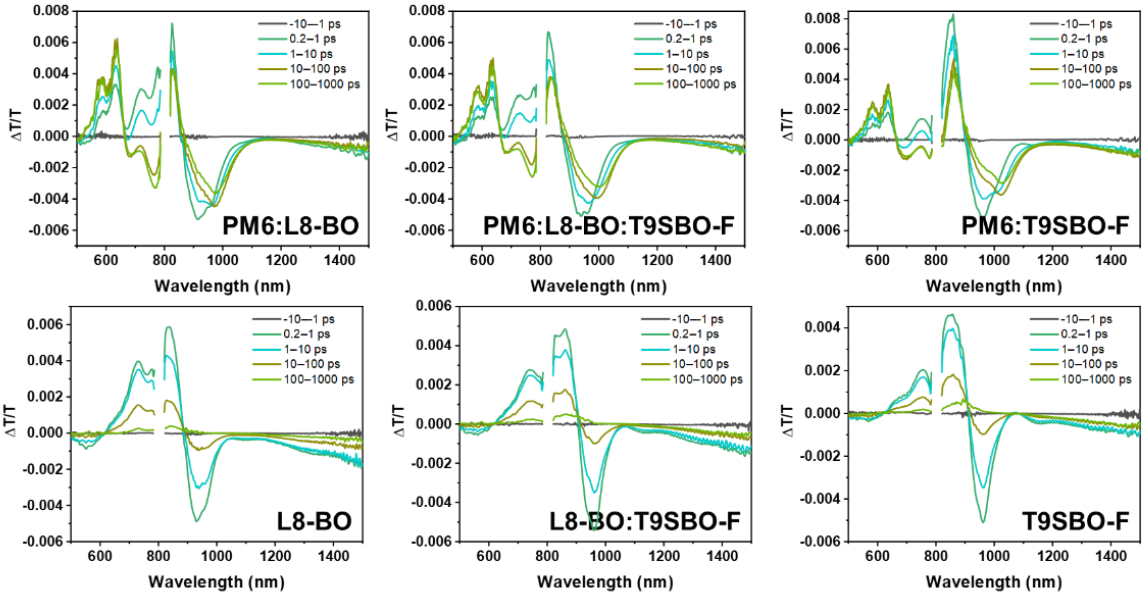

Supplementary Fig. 7. TA spectra of blend and neat acceptor films. The samples are pumped at 800 nm to excite the acceptors.

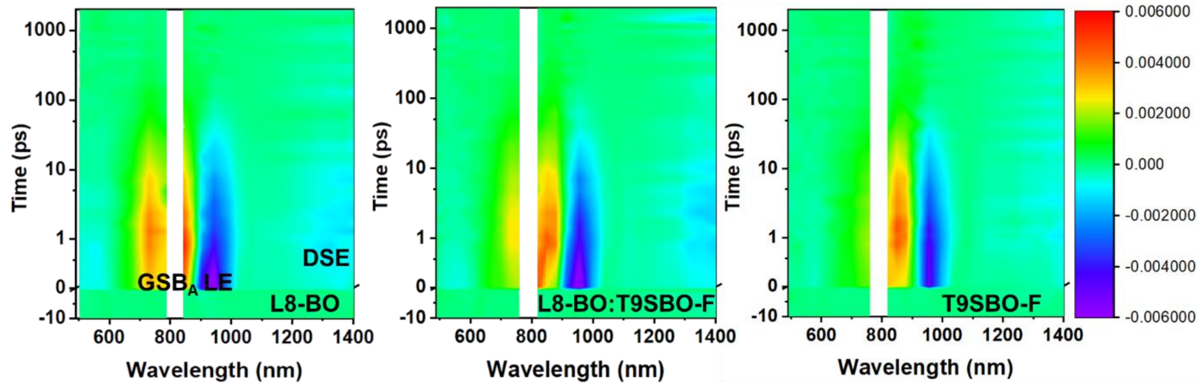

Supplementary Fig. 8. 2D TA contour plot of neat acceptor films. The samples are pumped at 800 nm to excite the acceptors.

Supplementary Table 3. Hole-transfer time. The bi-exponential fitting results of the donor ground-state bleach signal.

	PM6:L8-BO	PM6:L8-BO:T9SBO-F	PM6:T9SBO-F
τ_1 (ps)	1.18	0.89	0.49
τ_2 (ps)	9.15	6.21	4.83

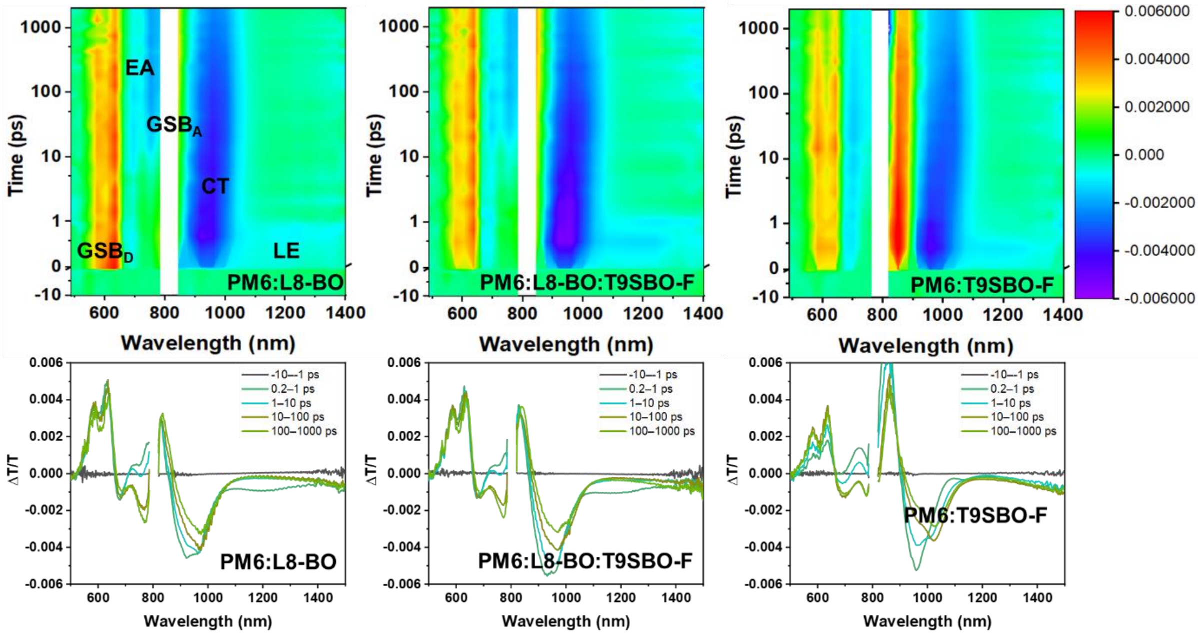

Supplementary Fig. 9. TA results of the blend films. The samples are pumped at 550 nm to excite donor PM6.

Supplementary Note IV

FTPS-EQE was performed on a Vertex Optics with an external detector option. The photocurrent of devices is amplified by an SR570 amplifier and the light source is modulated by a Fourier transform infrared (FTIR) spectroscope. EL measurement was conduced on a Shamrock SR-303i spectrometer from Andor Tech with a Newton EM-CCD Si. Voltage bias is applied on the samples via a Keithley 2400 source meter. EQE_{EL} is monitored by an home-built system with a Hamamatsu silicon photodiode 1010B, a Keithley 2400 source meter and a Keithley 485 Picoammeter to measure the emitted light intensity.

The energy loss is quantified by the following equation:[4-6]

$$\Delta E = \Delta E_1 + \Delta E_2 + \Delta E_3 = (E_g - qV_{OC}^{SQ}) + (qV_{OC}^{SQ} - qV_{OC}^{rad}) + (qV_{OC}^{rad} - qV_{OC}) \quad (4)$$

The first energy loss term (ΔE_1) is the energy difference between bandgap and Shockley-Queisser (SQ) limit, which depends on the bandgap and normally resides in 0.25-0.3 eV for photovoltaic devices. qV_{OC}^{SQ} can be calculated by the following equation:

$$qV_{OC}^{SQ} = k_B T \ln\left[\frac{q\int_{E_g}^{\infty} \emptyset_{\mathrm{AM1.5G}}(E)dE}{q\int_{E_g}^{\infty} \emptyset_{\mathrm{BB}}(E)dE} + 1\right] \quad (5)$$

where k_B is Boltzmann constant, T is absolute temperature and $\emptyset_{\mathrm{AM1.5G}}(E)$ is the irradiance of solar light under AM 1.5G condition. $\emptyset_{\mathrm{BB}}(E)$ is the blackbody spectrum of the semiconductors, which is calculated by:[5,7]

$$\emptyset_{\mathrm{BB}}(E) = \frac{2\pi E^2}{h^3 c^2} \exp\left(-\frac{E}{k_B T}\right) \quad (6)$$

where h is Planck constant and c is the light speed.

ΔE_2 describes the radiative energy loss below the bandgap. qV_{OC}^{rad} can be obtained by:

$$qV_{OC}^{rad} = k_B T \ln\left[\frac{Jsc}{q\int_0^{\infty} \eta_{\mathrm{EQE}}(E)\phi_{\mathrm{BB}}(E)dE} + 1\right] \quad (7)$$

The final term (ΔE_3) represents the non-radiative recombination loss correlating to the external radiative efficiency ($\mathrm{EQE_{EL}}$).

$$\Delta E_3 = -k_B T \ln(\mathrm{EQE_{EL}}) \quad (8)$$

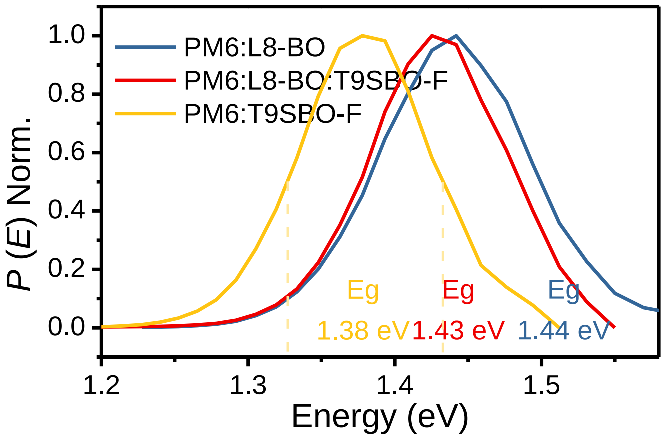

Supplementary Fig. 10. Optical bandgap determination. The distribution of SQ-type bandgaps derived from $\mathrm{EQE_{ftps}}$ ($P(E) = \mathrm{dEQE_{ftps}}/\mathrm{d}E$).

Supplementary Table 4. Energy loss of the devices.

	E_{gap} (eV)	qV_{oc} (eV)	E_{loss} (eV)	V_{oc}^{SQ} (V)	ΔE_1 (eV)	V_{oc}^{rad} (V)	ΔE_2 (eV)	ΔE_3 (eV)
PM6:L8-BO	1.440	0.888	0.552	1.175	0.265	1.123	0.052	0.232
PM6:L8-BO:T9SBO-F	1.430	0.881	0.549	1.166	0.264	1.107	0.059	0.221
PM6:T9SBO-F	1.380	0.829	0.551	1.119	0.261	1.066	0.052	0.233

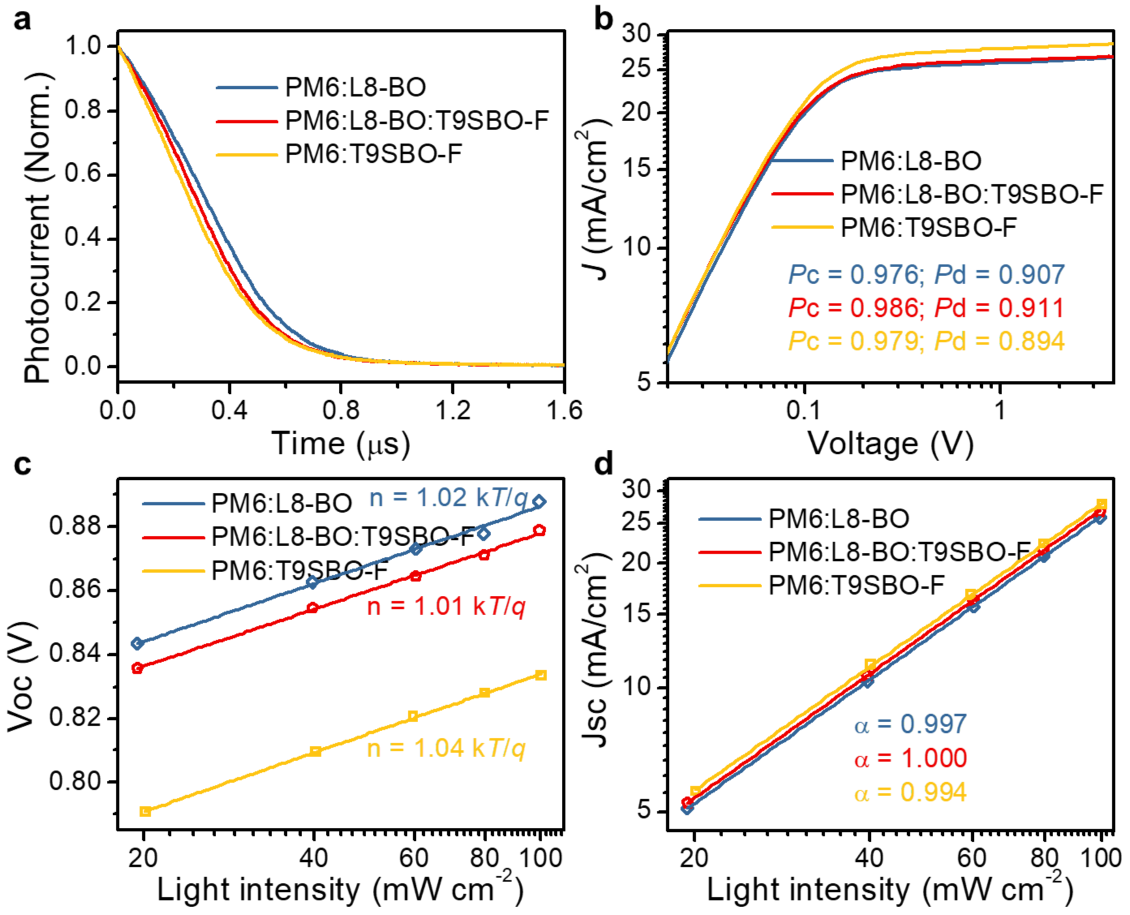

Supplementary Fig. 11. Recombination loss analysis. (a) TPC measurement and (b) photocurrent verses effective voltage curves of different devices; (c) V_{OC} and (d) J_{SC} under different light intensities.

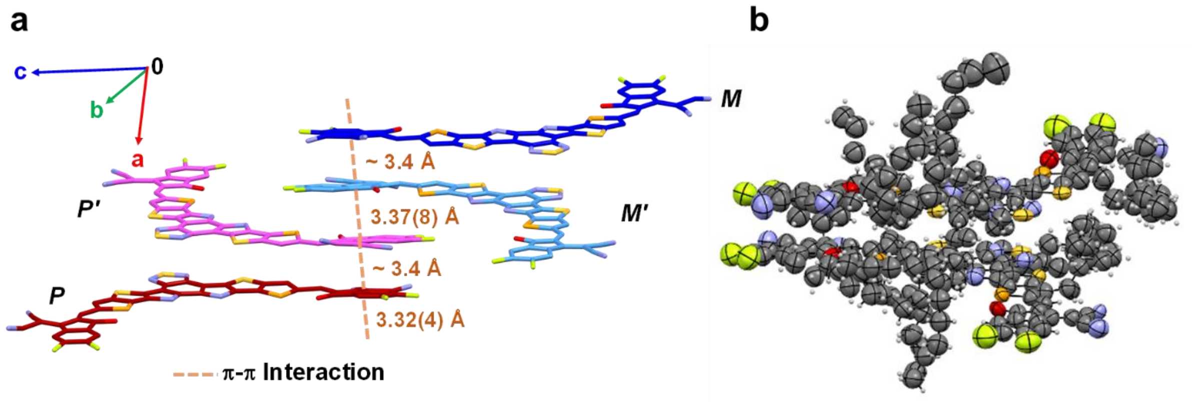

Supplementary Fig. 12. Single crystal information. (a) Molecular packing with four enantiomers in the T9SBO-F single crystal. (b) The Oak Ridge Thermal Ellipsoid Plot (ORTEP) view of T9SBO-F with atomic displacement parameters shown at the 50% probability level.

Notably, the single crystal CheckCif indicates A- and B-level alerts as shown below:

Alert Level A
EXPT005_ALERT_1_A _exptl_crystal_description is missing
Crystal habit description.
The following tests will not be performed. CRYSR_01
DIFF003_ALERT_1_A _diffrn_measurement_device_type is missing
Diffractometer make and type. Replaces _diffrn_measurement_type.
THETM01_ALERT_3_A The value of sine(theta_max)/wavelength is less than 0.550
Calculated sin(theta_max)/wavelength = 0.4166
PLAT029_ALERT_3_A _diffrn_measured_fraction_theta_full value Low. 0.884
PLAT088_ALERT_3_A Poor Data / Parameter Ratio ... 4.76 Note
PLAT183_ALERT_1_A Missing _cell_measurement_reflns_used Value
PLAT184_ALERT_1_A Missing _cell_measurement_theta_min Value
PLAT185_ALERT_1_A Missing _cell_measurement_theta_max Value
PLAT699_ALERT_1_A Missing _exptl_crystal_description Value
Alert Level B
PLAT082_ALERT_2_B High R1 Value. 0.19 Report
PLAT084_ALERT_3_B High wR2 Value (i.e. > 0.25). 0.44 Report
PLAT341_ALERT_3_B Low Bond Precision on C-C Bonds. 0.04037 Ang.
PLAT911_ALERT_3_B Missing FCF Refl Between Thmin & STh/L= 0.417. 1286 Report

Alerts justification: The crystal is labile and hard to resolve due to the high degree of disorder arisen from the long alkyl chains and loss of solvent molecules during measurements. Therefore, the collected diffraction data is not of the best quality and thus level A- and B-alerts are shown in the CheckCIF report. However, in the work, we only made analysis based on the conjugated parts of the molecule, not the alkyl chains. The results from our analysis are also consistent with our previously published works (CH1007: CCDC 2009659;[8] T9TBO-F: CCDC 2081901, α-T9SBN-F: CCDC 2084244 and β-T9SBN-F: CCDC 2081902[9]).

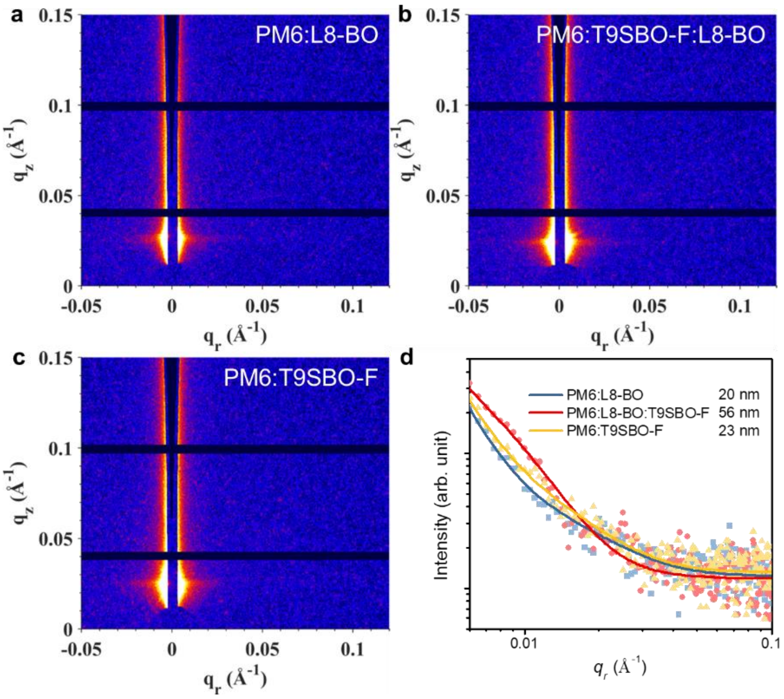

Supplementary Fig. 13. Nanoscale phase separation. 2D GISAXS pattern of (a) PM6:L8-BO (b) PM6:L8-BO:T9SBO-F and (c) PM6:T9SBO-F film. (d) The 1D line-cut in-plane GISAXS profile and the fitted results.

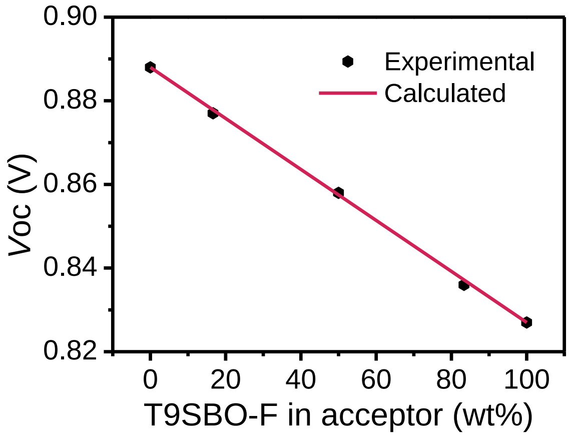

Supplementary Fig. 14. *V*oc dependency on the weight ratio of T9SBO-F in the acceptor. The value is obtained from at least 10 individual devices.

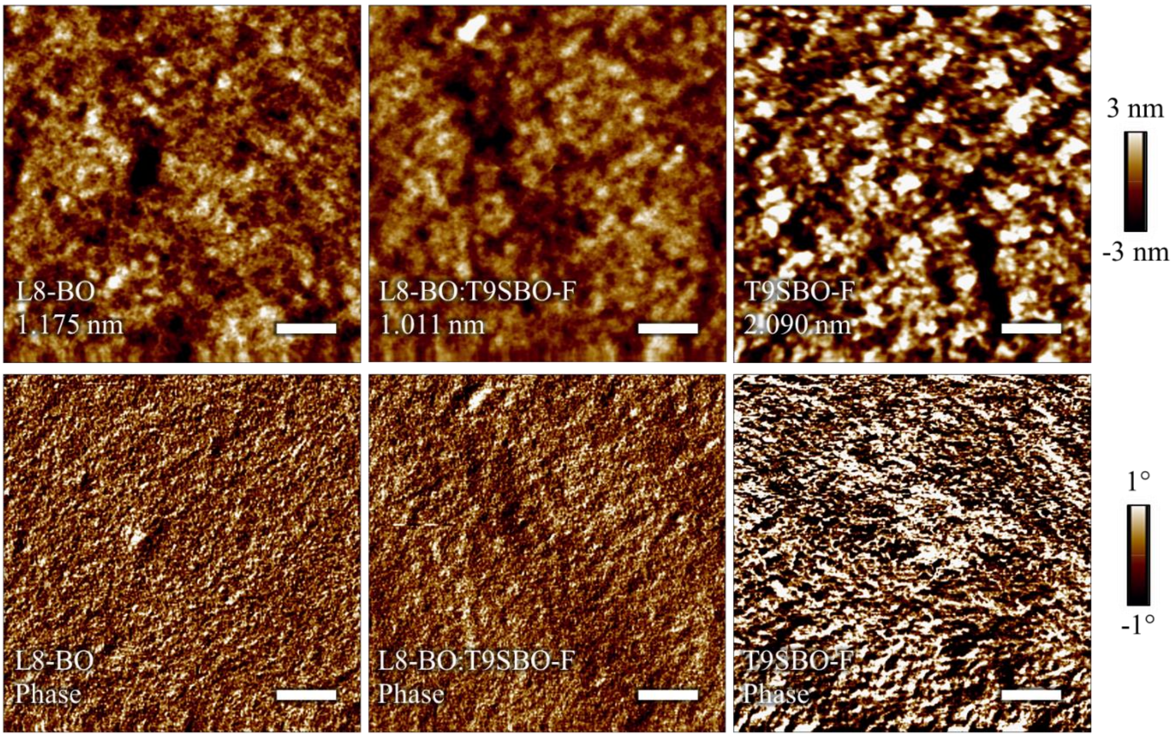

Supplementary Fig. 15. AFM images of donor:acceptor blend samples. The scale bar is 500 nm.

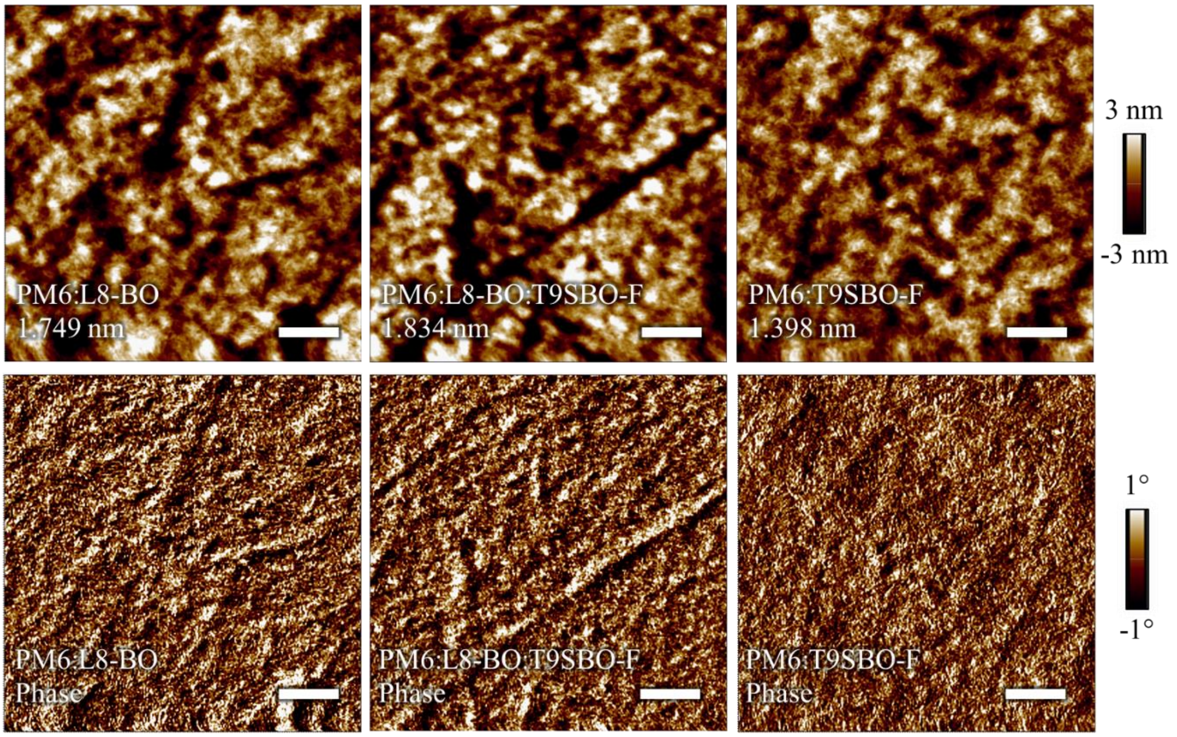

Supplementary Fig. 16. AFM images of neat acceptor samples. The scale bar is 500 nm.

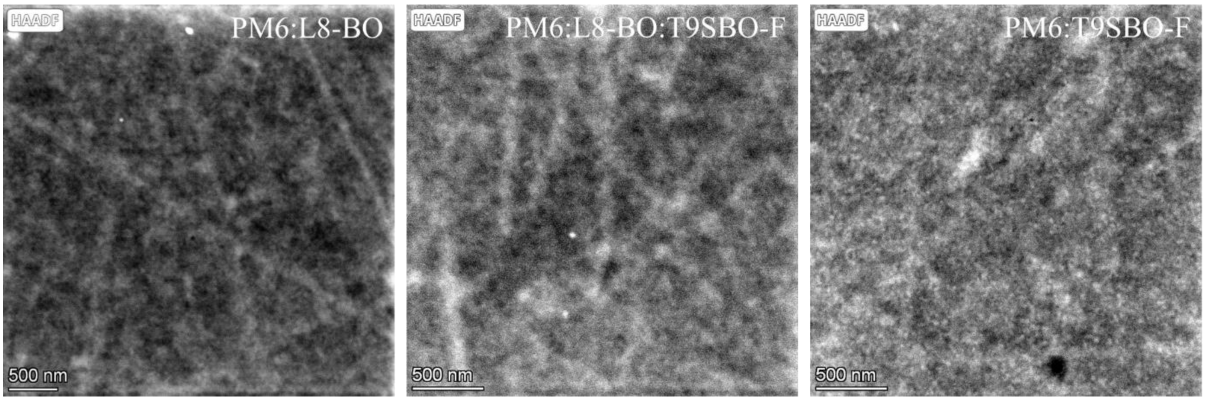

Supplementary Fig. 17. HAADF-STEM images of donor:acceptor blend samples.

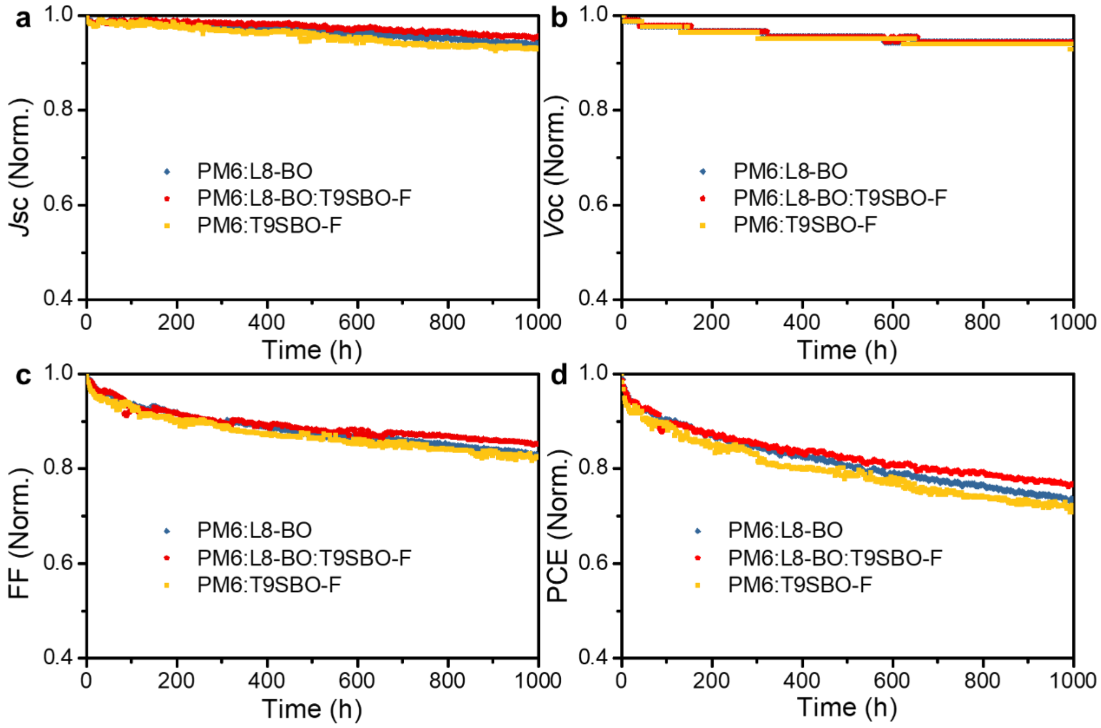

Supplementary Fig. 18. Stability measurement. (a) *J*sc (b) *V*oc (c) FF and (d) PCE of the encapsulated devices in ambient condition with MPP tracking and continuous LED light illumination (100 mW/cm^2).

Supplementary References

1 Li, T. *et al.* Asymmetric Glycolated Substitution for Enhanced Permittivity and Ecocompatibility of High-Performance Photovoltaic Electron Acceptor. *JACS Au* **1**, 1733-1742, (2021).

2 Su, R. *et al.* Dielectric screening in perovskite photovoltaics. *Nat. Commun.* **12**, 2479, (2021).

3 Awni, R. A. *et al.* Influence of Charge Transport Layers on Capacitance Measured in Halide Perovskite Solar Cells. *Joule* **4**, 644-657, (2020).

4 Liu, S. *et al.* High-efficiency organic solar cells with low non-radiative recombination loss and low energetic disorder. *Nat. Photon.* **14**, 300-305, (2020).

5 Sun, C. *et al.* Dimerized small-molecule acceptors enable efficient and stable organic solar cells. *Joule,* (2023).

6 Li, C. *et al.* Non-fullerene acceptors with branched side chains and improved molecular packing to exceed 18% efficiency in organic solar cells. *Nat. Energy* **6**, 605-613, (2021).

7 Rau, U., Blank, B., Müller, T. C. M. & Kirchartz, T. Efficiency potential of photovoltaic materials and devices unveiled by detailed-balance analysis. *Phys. Rev. Appl.* **7**, 044016, (2017).

8 Lin, F., Jiang, K., Kaminsky, W., Zhu, Z. & Jen, A. K. Y. A Non-fullerene Acceptor with Enhanced Intermolecular π-Core Interaction for High-Performance Organic Solar Cells. *J. Am. Chem. Soc.* **142**, 15246-15251, (2020).

9 Jiang, K. *et al.* Suppressed recombination loss in organic photovoltaics adopting a planar–mixed heterojunction architecture. *Nat. Energy* **7**, 1076-1086, (2022).
