# Supplementary material for: Selenium substitution for dielectric constant improvement and hole-transfer acceleration in non-fullerene organic solar cells
Source: Nat Commun. 2024 Mar 7;15:2103. doi: 10.1038/s41467-024-46352-2 (PMC10920633; doi:10.1038/s41467-024-46352-2)
Supplement: Supplementary file 3 — Solar Cells Reporting Summary [file 41467_2024_46352_MOESM3_ESM.pdf]

Corresponding author(s): Wallace C.H. Choy, Alex K.-Y. Jen, Kam Sing Wong

Solar Cells Reporting Summary

Nature Portfolio wishes to improve the reproducibility of the work that we publish. This form is intended for publication with all accepted papers reporting the characterization of photovoltaic devices and provides structure for consistency and transparency in reporting. Some list items might not apply to an individual manuscript, but all fields must be completed for clarity.

For further information on Nature Research policies, including our data availability policy, see Authors & Referees.

Experimental design

Please check the following details are reported in the manuscript, and provide a brief description or explanation where applicable.

1. Dimensions

Item	Yes/No	Description
Area of the tested solar cells	[x] Yes	The device active area is 0.075 mm2, the aperture area is 0.05 mm2.
	[ ] No	*Explain why this information is not reported/not relevant.*
Method used to determine the device area	[x] Yes	The aperture area of the shadow mask was measured by vernier caliper and optical microscope.
	[ ] No	*Explain why this information is not reported/not relevant.*

2. Current-voltage characterization

Item	Yes/No	Description
Current density-voltage (J-V) plots in both forward and backward direction	[ ] Yes	
	[x] No	Considering organic solar cells do not have hysteresis issue, we only performed forward scan on the devices.
Voltage scan conditions	[x] Yes	The devices were scanned in forward direction from -0.1 to 0.9 or 0.95 V with a scan step of 0.01 V and dwell time of 10 ms.
	[ ] No	*Explain why this information is not reported/not relevant.*
Test environment	[x] Yes	The devices were tested in N2-filled glove box at room temperature.
	[ ] No	*Explain why this information is not reported/not relevant.*
Protocol for preconditioning of the device before its characterization	[ ] Yes	*Provide a description of the protocol.*
	[x] No	No preconditioning protocol was performed.
Stability of the J-V characteristic	[ ] Yes	*Provide a description of the method used. The stability of the J-V characteristic can be verified with time evolution of the maximum power point or with the photocurrent at maximum power point; see ref. 5 for details.*
	[x] No	We only tested the light stability in our lab.

3. Hysteresis or any other unusual behaviour

Item	Yes/No	Description
Description of the unusual behaviour observed during the characterization	[ ] Yes	*Provide a description of hysteresis or any other unusual behaviour observed during the characterization.*
	[x] No	No hysteresis or unusual behaviour was observed during the characterization of the solar cells.
Related experimental data	[ ] Yes	*Provide a description of the related experimental data.*
	[x] No	No hysteresis or unusual behaviour was observed during the characterization of the solar cells.

4. Efficiency

Item	Yes/No	Description
External quantum efficiency (EQE) or incident photons to current efficiency (IPCE)	[x] Yes	The EQE spectra were measured using an Enlitech QE-R EQE system with a standard Si detector, as shown in Figure 1f.
	[ ] No	*Explain why this information is not reported/not relevant.*

A comparison between the integrated response under the standard reference spectrum and the response measure under the simulator

- [x] Yes
- [ ] No

The integrated Jsc from EQE agreed well with the Jsc measured from J-V measurement (less than 6% mismatch.)

Explain why this information is not reported/not relevant.

For tandem solar cells, the bias illumination and bias voltage used for each subcell

- [ ] Yes
- [x] No

Provide a description of the measurement conditions.

No tandem solar cell is reported in this work.

5. Calibration

Light source and reference cell or sensor used for the characterization

- [x] Yes
- [ ] No

Enlitech AAA solar simulator (SS-F5-3A) and standard Si (SRC-2020) reference cell were used for the solar cell measurement (see Methods)

Explain why this information is not reported/not relevant.

Confirmation that the reference cell was calibrated and certified

- [x] Yes
- [ ] No

The reference cell (SRC-2020) was calibrated by NREL.

Explain why this information is not reported/not relevant.

Calculation of spectral mismatch between the reference cell and the devices under test

- [ ] Yes
- [x] No

Provide a value of the spectral mismatch and/or a description of how it has been taken into account in the measurements.

No spectral mismatch calculation was performed in our lab.

6. Mask/aperture

Size of the mask/aperture used during testing

- [x] Yes
- [ ] No

A 0.05 cm2 aperture shadow mask was employed to define the device area. The aperture area was measured by vernier caliper and optical microscope.

Explain why this information is not reported/not relevant.

Variation of the measured short-circuit current density with the mask/aperture area

- [ ] Yes
- [x] No

Report the difference in the short-circuit current density values measured with the mask and aperture area.

For the cells tested in our lab, they all used the same shadow mask.

7. Performance certification

Identity of the independent certification laboratory that confirmed the photovoltaic performance

- [ ] Yes
- [x] No

Identify the independent certification laboratory.

We did not perform certification on the solar cells.

A copy of any certificate(s)

- [ ] Yes
- [x] No

Certificate copies should be provided in the Supplementary information. Please state the supplementary item number.

We did not perform certification on the solar cells.

8. Statistics

Number of solar cells tested

- [x] Yes
- [ ] No

At least 10 individual devices were tested for each condition.

Explain why this information is not reported/not relevant.

Statistical analysis of the device performance

- [x] Yes
- [ ] No

Please see Fig. 1e, Table 1 and Supplementary Table 2.

Explain why this information is not reported/not relevant.

9. Long-term stability analysis

Type of analysis, bias conditions and environmental conditions

- [x] Yes
- [ ] No

Please see Supplementary Fig. 18. The devices are encapsulated and tested in ambient conditions with MPP tracking and continuous LED light illumination (100 mW/cm2)

Explain why this information is not reported/not relevant.
